# Safety and Feasibility of Coronary Stenting in Unprotected Left Main Coronary Artery Disease in the Real World Clinical Practice—A Single Center Experience

**DOI:** 10.1371/journal.pone.0109281

**Published:** 2014-10-20

**Authors:** Wei-Chieh Lee, Tzu-Hsien Tsai, Yung-Lung Chen, Cheng-Hsu Yang, Shyh-Ming Chen, Chien-Jen Chen, Cheng-Jei Lin, Cheng-I Cheng, Chi-Ling Hang, Chiung-Jen Wu, Hon-Kan Yip

**Affiliations:** 1 Division of Cardiology, Department of Internal Medicine, Kaohsiung Chang Gung Memorial Hospital and Chang Gung University College of Medicine, Kaohsiung, Taiwan; 2 Center for Translational Research in Biomedical Sciences, Kaohsiung Chang Gung Memorial Hospital and Chang Gung University College of Medicine, Kaohsiung, Taiwan; 3 Institute of Shock Wave Medicine and Tissue Engineering, Kaohsiung Chang Gung Memorial Hospital and Chang Gung University College of Medicine, Kaohsiung, Taiwan; University of Washington, United States of America

## Abstract

**Background:**

This study evaluated the feasibility, safety, and prognostic outcome in patients with significant unprotected left main coronary artery (ULMCA) disease undergoing stenting.

**Method and Results:**

Between January 2010 and December 2012, totally 309 patients, including those with stable angina [13.9% (43/309)], unstable angina [59.2% (183/309)], acute non-ST-segment elevation myocardial infarction (NSTEMI) [24.3% (75/309)], and post-STEMI angina (i.e., onset of STEMI<7 days) [2.6% (8/309)] with significant ULMCA disease (>50%) undergoing stenting using transradial arterial approach, were consecutively enrolled. The patients’ mean age was 68.9±10.8 yrs. Incidences of advance congestive heart failure (CHF) (defined as ≥ NYHA Fc 3) and multi-vessel disease were 16.5% (51/309) and 80.6% (249/309), respectively. Mechanical supports, including IABP for critical patients (defined as LVEF <35%, advanced CHF, or hemodynamically unstable) and extra-corporeal membrane oxygenator (ECMO) for hemodynamically collapsed patients, were utilized in 17.2% (53/309) and 2.6% (8/409) patients, respectively. Stent implantation was successfully performed in all patients. Thirty-day mortality rate was 4.5% (14/309) [cardiac death: 2.9% (9/309) vs. non-cardiac death: 1.6% (5/309)] without significant difference among four groups [2.3% (1) vs. 2.7% (5) vs. 9.3% (7) vs. 12.5% (1), p = 0.071]. Multivariate analysis identified acute kidney injury (AKI) as the strongest independent predictor of 30-day mortality (p<0.0001), while body mass index (BMI) and white blood cell (WBC) count were independently predictive of 30-day mortality (p = 0.003 and 0.012, respectively).

**Conclusion:**

Catheter-based LM stenting demonstrated high rates of procedural success and excellent 30-day clinical outcomes. AKI, BMI, and WBC count were significantly and independently predictive of 30-day mortality.

## Introduction

Previous studies have revealed that medically treated patients with significantly unprotected left main coronary artery (ULMCA) disease (i.e., >50% stensosis) have a 3-year mortality rate up to 50% [1], [2]. Several clinical trials have shown a superior survival benefit of coronary bypass grafting (CABG) compared with medical treatment for significant ULMCA disease [3]–[6]. Based on the evidence of these trials [3]–[6], the current practice guideline still recommends CABG as the gold standard for the treatment of significant left main coronary artery (LM) disease [7], [8]. However, several points have to be taken into consideration. Despite a well-established technique, CABG is a major surgical procedure associated with significant operative risk and up to 3.0% in-hospital mortality [9]. Moreover, CABG carries an especially high risk or is not feasible in patients with 1) advanced age or critical internal medical co-morbidities, 2) short estimated life expectancy as in those with malignancies, 3) significant ULMCA disease with urgent requirement for major non-cardiovascular surgical intervention, 4) low willingness to receive CABG, or 5) unstable condition/hemodynamic collapse due to an acute LM occlusion. Another therapeutic option other than CABG or medical treatment, therefore, is of utmost importance to physicians.

Over the last 20 years, with the accumulation of operators’ experience, refinement in instruments, and advance in pharmacological development of anti-platelet and anti-ischemic agents, percutaneous coronary intervention (PCI) has been widely accepted as one of the most popular methods for the treatment of atherosclerotic occlusive syndrome, especially for patients with ST-segment elevation myocardial infarction (STEMI) with or without cardiogenic shock [10], [11]. These advances in PCI techniques and stent technology have allowed evaluation of the role of PCI in significant ULMCA disease [12], [13], especially focusing on the safety and efficacy of stenting the LMCA to determine whether it does provide a true alternative to CABG [12]–[15]. Results from previous clinical trials comparing the efficacy and safety between PCIs with stenting and CABG have shown comparable results in terms of procedural success rates, safety, favorable early outcomes, and the need for repeated revascularization [12]–[19]. However, many data were from clinical trials with strict exclusion criteria for patient selection [12]–[15], [17]–[19] rather than a real-world clinical practice [16] without patient exclusion. Of importance is that patients with unstable clinical presentation, hemodynamic compromise upon presentation, or patients in the setting of acute or early myocardial infarction were usually excluded from the trials [12]–[15], [17]–[19]. Thus, further evidence-based information should be acquired to assess the lay the clinical foundation for the practice of LMCA stenting [18]. The issue is of particular importance in Asia where the majority of patients are unwilling to receive CABG due to a fear for chest surgery based on a traditional belief, making PCI the last resort for the treatment of significant LM disease. Accordingly, this study, based on the needs arising from our daily clinical practice for clarifying the safety, feasibility, and assessing the 30-day clinical outcome of patients with significant ULMCA disease undergoing PCI because of refusal of CABG or unsuitability for surgery, attempted to evaluate the benefit of mechanical-assisted procedure to patients in critical condition upon presentation.

## Materials and Methods

### Patient Population, Ethics, Enrollment and Exclusion Criteria

This study was approved by the Institutional Review Committee on Human Research of Chang Gung Memorial Hospital (No 102-0789B) and conducted at Kaohsiung Chang Gung Memorial Hospital for retrospective assessment of procedural success rate, safety, efficacy, and 30-day clinical outcomes in patients with significant ULMCA disease with clinical presentations as (1) stable angina pectoris (SAP) (group 1), (2) unstable angina pectoris (UAP) (group 2), (3) acute or recent (i.e., onset < day 7) non-ST elevation myocardial infarction (NSTEMI) (group 3), and (4) post-ST-segment elevation myocardial infarction (STEMI) angina (i.e., >12 h <7-day presentation of STEMI) (group 4). The participants provide their written informed consent to participate in this study. The ethics committees approve this consent procedure.

Our daily clinical practice in Kaohsiung Chang Gung Memorial Hospital is still according to the current guideline for treatment of significant ULMCA disease [7], [8]. Accordingly, if patients with angiographic findings of significant ULCMA in the setting of STEMI, immediate primary PCI/stenting was done for the patients without hesitation, especially for those with unstable hemodynamics. Thus, patients experienced acute LM occlusion resulted from acute STEMI were excluded from the study. On the other hand, if patients with angiographic findings of significant ULCMA in the settings of SAP, UAP, STEMI and post-STEMI angina, we (i.e., both interventional cardiologist and surgeon) fully explained to patients and recommended CABG as the treatment of choice for the disease. However, PCI for significant ULMCA disease was performed in the following situations: 1) Patients refused to receive CABG treatment; 2) Advanced age or in patients with critical internal medical co-morbidities who were unwilling to receive CABG; 3) Estimated life expectancy is short as in those with known malignancy; 4) Treatment of significant ULMCA disease as a bridge to enable urgent non-cardiovascular major surgical intervention; 5) Patients in unstable condition/hemodynamic collapse during cardiac catheterization due to significant ULMCA disease, or post-CABG with occlusion of left internal mammary artery (LIMA) to left anterior descending artery (LAD) and multiple vessel disease.

Between January 2010 and December 2012, totally 342 patients, including those with SAP [13.9% (43/309)], UAP [59.2% (183/309)], NSTEMI [24.3% (75/309)], and post-STEMI angina (i.e., onset of STEMI <7 days) [2.6% (8/309)], undergoing PCI/stenting for the significant ULMCA disease were retrospectively enrolled in the current study. Informed consent was obtained from each study subject. The Institutional Review Committee on Human Research at our institution approved the study protocol.

### Procedure and Protocol of Cardiac Catheterization Approach and Indications of Intra-Aortic Balloon Pump Support

For elective or primary PCI, a transradial artery approach using a 6-French arterial sheath is a routine procedure for patients at Kaohsiung Chang Gang Memorial Hospital unless Allen’s test is positive on both sides. Routine trans-radial or trans-brachial arterial approach is also adopted in each patient for LM stenting/PCI using 6-French (F) or 7-F guiding catheter dependent on the LM lesion character and the strategy of stent implantation. Additionally, other vessels with significant obstruction that limited the blood flow were eligible for PCI at the same stage or during hospitalization. The detailed procedure and protocol have been reported in our previous studies [10], [11], [20], [21].

Intra-aortic balloon pump (IABP) support was performed via right femoral arterial approach in patients experiencing advanced congestive heart failure (CHF) [defined as New York Heart Association Function classification (Fc)≧Fc III], or acute pulmonary edema associated with unstable condition or hemodynamic instability. Moreover, elective IABP support (i.e., as a bridge of mechanical support) prior to LM PCI was performed for patients with severe left ventricular dysfunction [i.e., left ventricular ejection fraction (LVEF) <35%] without CHF. IABP was promptly removed in patients after LM PCI.

### Definitions of Cardiogenic Shock and Profound Shock and Criteria for Extra-Corporeal Membrane Oxygenator Support

Definitions of cardiogenic shock and profound shock were based on our previous report [10], [11], [22]. Briefly, patients who experienced cardiogenic shock upon presentation or were observed at catheterization room met the following prospectively defined criteria for early cardiogenic shock: (1) Chest x-ray showing pulmonary edema with systolic blood pressure (SBP) <90 mmHg, or (2) Persistent hypotension with SBP<90 mmHg associated with low cardiac output and clear lung fields, not related to dysrhythmia, showing no response to adequate fluid supply, and requiring vasopressor agent infusion. In addition, profound shock was defined as SBP<75 mmHg despite intravenous inotropic agent administration and IABP support associated with altered mental status and respiratory failure.

Extra-corporeal membrane oxygenator (ECMO) was inserted at catheterization room for patients whose SBP could not be maintained above 75 mmHg after IABP support and intravenous administration of dopamine >20 g/kg/min.

### Functional Assessment by Echocardiography

LV function was assessed using transthoracic echocardiography. With the patients in a supine position, left ventricular internal dimensions [i.e. end-systolic diameter (ESD) and end-diastolic diameter (EDD)] were measured according to the American Society of Echocardiography leading-edge method using at least 3 consecutives cardiac cycles. The LV ejection fraction (LVEF) was calculated as: LVEF (%) = [(LVEDD^3^−LVEDS^3^)/LVEDD^3^]×100.

### Angiographic Analysis and Definitions

Quantitative angiographic analysis of the degree of coronary artery luminal stenosis and the reference lumen diameter was conducted using a digital edge-detection algorithm (DUQUE System) by selecting end-diastolic frames to demonstrate stenosis in its most severe and non-foreshortened projection [23]. With the contrast-filled guiding catheter serving as the calibration standard, the reference and minimal lumen diameters were determined before and after angioplasty. Single-vessel disease was defined as stenoses of >50% in 1 major epicardial coronary artery. Multi-vessel disease was defined as stenoses of >50% in ≥2 major epicardial coronary arteries. Body mass index (BMI) was defined as body weight in kilograms divided by the square of body height in meters (kg/m^2^).

### Definitions

Procedure success was defined a final residual stenosis of less than 10% and normal blood flow was achieved in the LM coronary artery. The clinical success was defined as uneventful discharge within 30 days after the procedure. The feasibility was defined as failed procedure rate of less than 1.0%. Safety was defined as a rate of procedure-related major complication (i.e., failed procedure rate and procedure-related mortality) less than 1.0%.

Acute renal injury was defined as an elevation in serum creatinine level more than 0.5 mg/dL within 24 h.

### Medications

All patients received a loading dose of clopidogrel (600 mg orally) in the emergency room or at ward prior to cardiac catheterization, followed by a maintenance dose (75 mg/day orally once daily) for at least 9 months based on the current guideline after the procedure. Aspirin (100 mg orally once daily) was given indefinitely to each patient. Other commonly prescribed medications also included angiotensin-converting enzyme inhibitors, angiotensin II type I inhibitors, statins, beta-blockers, isonitrate, and diuretics.

### Data Collection and the End Points

For the purpose of the current study, all patients undergoing ULMCA PCI were retrospectively identified. Detailed in-hospital and follow-up data including age, gender, coronary risk factors, clinical condition on admission and during hospitalization, cardiac enzyme and creatinine level, New York Heart Association Functional Classification, number of diseased vessels, in-hospital adverse events, and 30-day mortality were obtained. In our hospital, we have had a program for catheter-based coronary intervention, including that of the primary percutaneous coronary intervention (primary PCI) since 31 may, 1993, the data, including those of the present study were collected prospectively and entered into a digital database consistently. The primary end point of this study was defined as the safety and efficacy of PCI. Secondary end point was defined as the 30-day survival rate.

### Statistical Analysis

Data were expressed as mean ± SD or % (number). Continue data which were expressed as mean ± SD were compared using one way ANOVA and followed by Bonferroni multiple-comparison post hoc test. Categorical data which were expressed as % (n) were analyzed by χ^2^ test and followed by Bonferroni multiple-comparison post hoc test. Univariate and multiple stepwise logistic regression analysis were used for determining the predictors of 30-day mortality. Statistical analysis was performed using SAS statistical software for Windows version 8.2 (SAS institute, Cary, NC). A P value of <0.05 was considered statistically significant.

## Results

### Baseline Characteristics of Four Groups (Table 1)

Table 1 shows the baseline characteristics of group 1 (SAP), group 2 (UAP), group 3 (NSTEMI), and group 4 (post-STEMI angina). The age was significantly higher in group 3 than that in groups 1, 2, and 4. Besides, the incidence of hypertension was significantly higher in group 4 than that in groups 1 to 3, but it showed no difference among the latter three groups. Furthermore, there were no significant differences in terms of male gender, body mass index, incidence of current smoking, diabetes mellitus, and serum level of total cholesterol, low-density lipoprotein, and high-density lipoprotein among the four groups. Moreover, the incidence of old myocardial infarction, previous stroke, chronic obstructive lung disease, previous PCI, or previous PCI for left main coronary artery disease also did not differ among four groups. However, the incidence of significant peripheral arterial obstructive disease was remarkably higher in groups 3 and 4 than that in groups 1 and 2.

**Table 1:** Baseline Characteristics of 309 Patients.

Variables	Group 1*(n = 43)	Group 2*(n = 183)	Group 3*(n = 75)	Group 4*(n = 8)	p-value
Age (yrs)	67.7±9.5^a^	67.5±10.5^a^	72.6±11.1^b^	71.1±14.5^a^	0.005
Male gender (%)	69.8% (30)	82% (150)	69.3% (52)	87.5% (7)	0.077
Body mass index (kg/m^2^)	25.4±3.6	24.8±3.6	25.0±4.1	24.9±3.5	0.850
Current smoking (%)	30.3% (13)	35.5% (65)	34.7% (26)	62.5% (5)	0.378
Diabetes mellitus (%)	51.2% (22)	46.4% (85)	42.7% (32)	75% (6)	0.333
Hypertension (%)	25.6% (11)^a^	18% (33)^a^	21.3% (16)^a^	50% (4)^b^	0.032
Total Cholesterol (mg/dL)†	156.9±53.6	162.9±48.7	161.8±39.4	147.3±30.5	0.732
LDL (mg/dL)†	91.6±40.9	90.1±41.7	96.2±35.9	85.3±23.1	0.699
HDL (mg/dL)†	48.0±16.7	45.9±16.5	44.7±13.7	43.8±13.5	0.724
Old myocardial infarction (%)	74.4% (32)	71% (130)	68% (51)	87.5% (7)	0.652
Previous CABG (%)	0% (0)	4.4% (8)	2.7% (2)	0% (0)	0.310
Previous PCI for LM (%)	11.6% (5)	11.5% (21)	2.7% (2)	0% (0)	0.104
History of COPD (%)	11.6% (5)	5.5% (10)	14.7% (11)	0% (0)	0.066
Symptomatic PAOD (%)	7% (3)^a^	4.4% (8)^a^	14.7% (11)^b^	12.5% (1)^b^	0.037
Old stroke (%)	7% (3)	11.5% (21)	20% (15)	12.5% (1)	0.170
ARB/ACEI (%)‡	48.8% (21)	48.1% (88)	36% (27)	37.5% (3)	0.308
Statin use (%)‡	51.2% (22)^a^	48.1% (88)^a^	30.7% (23)^b^	25% (2)^b^	0.033
Ac sugar (mg/dL)†	101.8±44.0^a^	112.4±56.7^a^	135.5±85.1^b^	106.1±22.9^a^	0.018
HBA1C (%)†	5.50±2.61	6.36±2.02	6.40±2.11	5.93±0.61	0.092
ESRD (%)	0% (0)^a^	8.7% (16)^b^	25.3% (19)^b^	0% (0)^a,b^	<0.001
Creatinine level (mg/dL)†	1.02±0.34^a^	1.92±2.60^a^	3.42±3.36^b^	1.86±2.23^a^	<0.001
White blood cell count†	7.2±2.6^a^	7.7±3.8^a^	9.7±3.4^b^	9.8±2.9^b^	<0.001
Troponin-I (ng/mL)†	0.3±0.9^a^	2.09±1.18^a^	45.3±183.1^b^	16.7±40.3^b^	0.001
CK-MB (unit/L)†	0.2±0.7^a^	0.39±2.3^a^	13.3±59.1^b^	7.43±20.6^b^	0.004
Troponin-I (after PCI) (ng/mL)	15.2±29.9^a^	15.2±40.6^a^	44.6±90.3^b^	44.1±60.2^b^	<0.001
CK-MB (after PCI) (unit/L)	3.16±7.13^a^	3.72±11.72^a^	18.9±37.4^b^	17.9±23.9^b^	<0.001

Data are expressed as % (n) or mean ± SD.

*Group 1 = angina pectoris, Group 2 = unstable angina, Group 3 = non ST-segment elevation myocardial infarction, Group 4 = post-ST-segment elevation myocardial infarction angina.

LDL = Low-density lipoprotein; HLD = high-density lipoprotein; CABG = coronary artery bypass surgery; LM = left main; PCI = percutaneous coronary intervention; COPD = chronic obstructive lung disease; PAOD = peripheral arterial obstructive disease; ARB/ACEI = angiotensin II type I receptor blcoker/angiotensin converting enzyme inhibitor; HBA1C = hemoglobin A1C; ESRD = end-stage renal disease; CK = Creatine phosphokinase.

† indicated measurement upon presentation.

‡ indicated therapy ≥5 week prior to be recorded.

Letters (^a,b^) indicate significant difference (at 0.05 level) by Bonferroni multiple-comparison post hoc test.

The incidence of the use of angiotensin II type I receptor blocker/angiotensin converting enzyme inhibitor (ARB/ACEI) was similar among the four groups. However, the incidence of stain use was significantly higher in groups 3 and 4 than in groups 1 and 2. Moreover, although the serum level of hemoglobin A1C (HBA1C) did not differ among the four groups, the plasma level of AC sugar was notably higher in group 3 than in groups 1, 2 and 4. Furthermore, the incidence of end stage renal disease (ESRD) was significantly higher in groups 2 and 3 than that in group 1. Moreover, the serum level of creatinine was markedly increased in group 3 than that in groups 1, 2, and 4.

The circulating levels of white blood cell (WBC) count, troponin-I, and creatine phosphokinase (CK)-MB upon admission or after PCI were significantly higher in groups 3 and 4 than those in groups 1 and 2.

### Clinical Presentation, Mechanical Supports, Heart Function, and Clinical Outcomes (Table 2)

Table 2 shows the clinically relevant factors and 30-day outcome in patients undergoing ULMCA PCI. The mean severity of CHF, the incidences of advanced CHF and acute respiratory failure were significantly higher in groups 3 and 4 than those in groups 1 and 2.

**Table 2:** Clinical Presentation, Heart Function, Incidence of Mechanical Supports, and 30-Day Clinical Outcome among 309 Patients.

Variables	Group 1*(n = 43)	Group 2*(n = 183)	Group 3*(n = 75)	Group 4*(n = 8)	p-value
Advanced CHF (≥Fc III) (%)†	14% (6)^a^	11.5% (21)^a^	26.7% (20)^b^	50% (4)^b^	0.001
Mean severity of CHF (mean ± SD)	0.21±0.68^a^	0.77±1.21^a^	1.60±1.54^b^	1.25±1.76^b^	<0.001
Acute respiratory failure (%)	2.3% (1)^a^	4.9% (9)^a^	26.7% (20)^b^	25% (2)^b^	<0.001
Systolic blood pressure (mmHg)‡	132±23	134±24	134±26	138±21	0.917
Diastolic blood pressure (mmHg)‡	71±13	72±15	71±15	78±25	0.709
Vasopressin agent use (%)§	4.7% (2)^a^	6% (11)^a^	32% (24)^b^	37.5% (3)^b^	<0.001
IABP support (%)¶	4.7% (2)^a^	10.4% (19)^a^	37.3% (28)^b^	50% (4)^b^	<0.001
ECMO support (%)ξ	0% (0)	2.2% (4)	5.3% (4)	0% (0)	0.294
LVEF (%)δ	55.1±25.8	56.7±20.4	53.1±14.6	55.6±12.7	0.611
Acute ischemic stroke (%)	0% (0)	0.5% (1)	1.3% (1)	0% (0)	0.823
Acute renal injury (%)	2.3% (1)	6% (11)	10.7% (8)	12.5% (1)	0.293
30-day mortality (%)	2.3% (1)	2.7% (5)	9.3% (7)	12.5% (1)	0.071
Hospital days (mean ± SD)	6.3±6.6^a^	8.6±15.6^a^	23.1±34.3^b^	15.1±10.9^b^	0.001

Data are expressed as % (n) or mean ± SD.

*Group 1 = angina pectoris, Group 2 = unstable angina, Group 3 = non ST-segment elevation myocardial infarction, Group 4 = post-ST-segment elevation myocardial infarction angina.

† defined as congestive heart failure (CHF) ≥ New York Heart Association Functional Classification (Fc) III.

‡ measured upon presentation.

§ intra-aortic balloon pump (IABP) was used for hypotension/shock.

¶ indication for poor left ventricular function [i.e., left ventricular function (LVEF) <35%], pulmonary edema, or hypotension/cardiogenic shock.

ξ extra-corporeal membrane oxygenator (ECMO) was used for profound cardiogenic shock.

δ indicated measurement of LVEF by transthoracic echocardiography.

Letters (^a,b^) indicate significant difference (at 0.05 level) by Bonferroni multiple-comparison post hoc test.

The mean systolic and diastolic blood pressures upon presentation did not differ among the four groups. In addition, the incidence of utilization of ECMO for profound cardiogenic shock did not differ among the four groups. However, the incidences of IABP support and utilization of inotropic agent for unstable hemodynamics were significantly higher in groups 3 and 4 than those in groups 1 and 2.

In addition to similarity in LVEF, the incidence of acute ischemic stroke also did not differ among four groups. However, the incidence of acute renal injury was significantly higher in groups 3 and 4 than in groups 1 and 2. Furthermore, the mean length of hospitalization showed an identical pattern compared to that of acute renal injury among the four groups. The overall 30-day mortality rate was 4.1% (14/342). There was no significant difference in the 30-day mortality among the four groups.

### Subgroup Analysis for the Cause of 30-Day Mortality, Comparison of BMI and Age between Survival and Dead Patients, and the Outcome of Mechanical Device Support (Table 3, Table 4 and Table 5)

**Table 3:** 30-Day Cardiac and Non-Cardiac Mortality among Four Groups.

Variables	Group 1	Group 2	Group 3	Group 4
30-day total mortality	1	5	7	1
Cardiac death	0	4	4	1
Non-cardiac death*	1	1	3	0

Group 1 = angina pectoris, Group 2 = unstable angina, Group 3 = non ST-segment elevation myocardial infarction, Group 4 = post-ST-segment elevation myocardial infarction angina.

*All non-cardiac death was due to sepsis.

**Table 4:** 30-Day Outcome of 8 Patients Supported by ECMO*.

Variables	Group 1	Group 2	Group 3	Group 4
No. of ECMO support patients	0	4	4	0
30-day total mortality	0	1	3	0
Cardiac death	0	1	2	0
Non-cardiac death†	0	0	1	0

ECMO = extra-corporeal membrane oxygenator.

*ECMO support for profound cardiogenic shock. These 8 patients also received intra-aortic balloon pump (IABP) support.

† death due to sepsis.

**Table 5:** 30-Day Outcome of 45 Patients Supported by IABP*.

Variables	Group 1	Group 2	Group 3	Group 4
No of IABP support patients	2	15	24	4
30-day total mortality	0	3	4	1
Cardiac death	0	2	2	1
Non-cardiac death*	0	1	2	0

IABP = intra-aortic balloon pump.

*death due to sepsis.

To further elucidate the causes of 30-day mortality, the database was carefully analyzed and the results showed that cardiac-related and non-cardiac-related death was 2.9% (9/309) and 1.6% (5/309). All the non-cardiac death was found to be related to sepsis after PCI. Additionally, the sepsis was suspected due to the implantations of catheters and or mechanical supports in the patient’s body. Further analysis revealed that the BMI (28.5±5.0 vs. 24.8±3.6, p<0.001) and the age (73.4±13.9 vs. 68.7±10.6, p = 0.016) was significantly increased in the 30-day dead patients compared to that in survival patients.

ECMO-assisted PCI was performed in 8 patients, including 4 patients in group 2 (i.e., UAP) and 4 patients in group 3 (i.e., NSTEMI), at cardiac catheterization room due to profound cardiogenic shock. The PCI procedure was successful in all 8 patients without procedure-related death. Four [50% (4/8)] patients with ECMO support were dead, including 1 in group 2 and 3 in group 3. Of these 4 fatalities, 3 were related to cardiac death and 1 was related to sepsis during hospitalization.

Isolated IABP support (i.e., without combined ECMO support) was given to 45 patients, including 2 in group 1, 15 in group 2, 24 in group 3, and 4 in group 4 at cardiac catheterization room due to poor LV function, hypotension/cardiogenic shock, advanced CHF, or acute pulmonary edema. The PCI procedure was successful in all 45 patients without procedure-related death. Mortality occurred in 4 patients [17.8% (8/45)] with IABP support, including 3 in group 2, 4 in group 3, and 1 in group 4. Of these 8 patients, cardiac death was implicated in 5 and 3 were related to sepsis during hospitalization.

In these IABP support patients, 14 of 45 patients (31.1%) of patients had received prophylactic IABP support for ULMCA stenting. The IABP support was promptly and successfully removed from all of the patients. All of these patients were uneventfully discharged from hospital.

### Angiographic Findings and PCI Results among Four Groups (Table 6)

Table 4 shows angiographic and PCI results among the study patients. The percentage of LM stenosis prior to PCI was significantly higher in groups 3 and 4 than that in groups 1 and 2. On the other hand, the incidences of multi-vessel disease, pre-PCI TIMI flow, and obstructive level and stenting position, including ostial/proximal, middle and distal/bifurcation portions, were similar among four groups. Moreover, pre-PCI and post-PCI minimal lumen diameter (MLD), reference lumen diameter, and post PCI residual stenosis were also similar among the four groups.

**Table 6:** Angiographic Findings and PCI Results among 342 Patients.

Variables	Group 1*(n = 43)	Group 2*(n = 183)	Group 3*(n = 75)	Group 4*(n = 8)	p-value
Multiple vessel disease (%)	79.1% (34)	79.8% (146)	81.3% (61)	100% (8)	0.553
Pre-PCI LM stenosis (%)	64.1±16.2^a^	67.5±12.3^a^	72.5±13.2^b^	73.9±5.1^b^	0.002
LM obstructive level					
Ostial/proximal level (%)	25.6% (11)	23.5% (43)	29.3% (22)	12.5% (1)	0.581
Middle level (%)	4.7% (2)	18% (33)	16% (12)	37.5% (3)	0.156
Distal/bifurcation level (%)	69.7% (30)	58.5% (107)	54.7% (41)	50% (4)	0.785
Pre-MLD (mm)	0.26±0.44	0.23±0.42	0.29±0.43	0.13±0.35	0.652
Pre-RLD (mm)	3.38±0.67	3.47±0.76	3.47±0.46	3.41±0.73	0.870
Pre-PCI TIMI flow					0.201
≥TIMI-2 flow	4.7% (2)	2.2% (4)	6.7% (5)	12.5% (1)	
≤TIMI-1 flow	95.3% (41)	97.8% (179)	93.3% (70)	87.5% (7)	
Stent position					
LM shaft (%)	14% (6)	15.3% (28)	16% (12)	0% (0)	0.665
LM-LAD (%)	58.1% (25)	54.1% (99)	53.3% (40)	62.5% (5)	0.922
LM-LCX (%)	7% (3)	7.7% (14)	4% (3)	0% (0)	0.675
LM-LAD-LCX (%)	20.9% (9)	23% (42)	26.7% (20)	37.5% (3)	0.653
Post-PCI LM stenosis (%)	2.95±0.21	2.95±0.33	2.89±0.42	2.87±0.35	0.539
Post-PCI MLD (mm)	3.39±0.59	3.39±0.61	3.77±0.67	3.27±0.77	0.387
Post-PCI RLD (mm)	3.38±0.58	3.48±0.60	3.91±0.64	3.49±0.44	0.863
Post-PCI TIMI-3 flow	100% (43)	100% (183)	100% (75)	100% (0)	1
Procedural success (%)	100% (43)	100% (183)	100% (75)	100% (8)	1
Type of stent implantation					0.021
Drug eluting stent	81.4% (35)^a^	91.8% (168)^b^	80% (60)^a^	100% (8)^a,b^	
Bare metal stenting	18.6% (8)^a^	8.2% (15)^b^	20% (15)^a^	0% (0)^a,b^	
Post stent dilatation (%)	100.0% (43)	100.0% (183)	100.0% (75)	100% (8)	1.0
IVUS examination (%)	67.4% (29)^a^	73.8% (135)^a^	49.3% (37)^b^	75% (6)^a,b^	0.002
No. PCI vessel (mean ± SD)	2.67±0.71	2.62±0.66	2.53±0.81	2.50±0.53	0.671
No. of stenting (mean ± SD)	2.34±1.15	2.32±1.11	2.40±1.01	3.12±0.76	0.252

Data are expressed as % (n) or mean ± SD.

*Group 1 = angina pectoris, Group 2 = unstable angina, Group 3 = non ST-segment elevation myocardial infarction, Group 4 = post-ST-segment elevation myocardial infarction angina.

PCI = percutaneous coronary intervention; LM = left main; MLD = minimal lumen diameter; RLD = reference lumen diameter; TIMI = thrombolisis in myocardial infarction; LAD = left anterior descending artery; LCX = left circumflex.

IVUS = intra-vascular ultra-sound.

Letters (^a,b^) indicate significant difference (at 0.05 level) by Bonferroni multiple-comparison post hoc test.

The achievement of final TIMI-3 flow and the procedural success rate did not differ among the four groups. Around 80% of all patients had received drug eluting stent implantation. The reasons for those 20% who did not receive drug eluting stent implantation were due to either economic problem or an LM diameter greater than 4.5 mm. The incidence of drug eluting stent use was significantly higher in group 2 than that in groups 1 and 3, but it showed no difference between groups 2 and 4 or among groups 1, 3, and 4. In addition, the incidence of bare metal stent implantation showed a reversed pattern compared to that of drug eluting stent implantation among the four groups. Furthermore, incidence of intra-vascular ultrasound (IVUS) utilization during the procedure showed a pattern identical to that of drug eluting stent use among the four groups. The overall utilization of the IVUS was only 60.5% (207/342) in the current study, mainly due to the economic problem. On the other hand, all of our patients had received high-pressure dilation after stent implantation. The number of PCI vessels and the number of stenting vessels did not differ among the four groups.

### Univariate and Multiple Stepwise Logistic Regression Analysis for 30-Day Mortality (Tables 7 and 8)

**Table 7:** Univariate Analysis for the Predictors of 30-Day Mortality.

Variables	Odds Ratio	95% CI	p-value
Age >70	4.466	1.221–16.339	0.024
Female gender	3.683	1.246–10.888	0.018
Body mass index*	1.261	1.105–1.437	0.001
Ac sugar*	1.007	1.001–1.013	0.025
White blood cell count (x10^3^)*	1.169	1.050–1.301	0.004
Acute kidney injury	20.071	6.185–65.132	<0.0001
Acute ischemic stroke	22.615	1.339–382.050	0.031

CI = confidence interval.

*indicated data were used as continuity.

**Table 8:** Multivariate Analysis for Independent Predictors of 30-Day Mortality.

Variables	Odds Ratio	95% CI	p-value
Body mass index*	1.243	1.079–1.431	0.003
White blood cell count (x10^3^)*	1.153	1.032–1.288	0.012
Acute kidney injury	16.040	3.667–70.162	<0.0001

CI = confidence interval.

*indicated data were used as continuity.

Table 5 shows the results of univariate analysis of the relevant variables in Table 1, 2, and 4 for predictors of 30-day mortality. The analytical results demonstrated that acute kidney injury was the first and body mass index was the second significantly strong predictor of 30-day mortality. In addition, WBC count, age, female gender, AC sugar, and acute ischemic stroke were also significantly associated with 30-day mortality.

Multiple stepwise logistic regression analysis demonstrated that acute kidney injury was the strongest independent predictor of 30-day mortality. Additionally, WBC count and BMI were significantly independent predictors of 30-day mortality.

## Discussion

This study which investigated the safety and feasibility of coronary stenting in patients with significant ULMCA stenosis yielded several striking clinical implications. First, the results of the present study demonstrated that catheter-based stenting for significant ULMCA disease was safe and feasible with very high procedural and clinical successful rates in real-world clinical practice. Second, in high-risk subgroup of patients and those patients with unstable condition, mechanical-assisted (i.e., IABP and ECMO) PCI for significant ULMCA disease offered an additional benefit with high rates of procedural success (i.e., 100%) and clinical success (95.5%). Third, in the relatively low-risk subgroups (i.e., SAP and UAP), the 30-day clinical outcome in the present study was not inferior to that previously reported for PCI or CABG clinical trials [6], [12]–[18] in a similar clinical setting of ULMAC disease.

### Real World Clinical Practice-Compliance to the Guideline and Flexible Utilization of Matured PCI Technique for Treating ULMCA disease

Despite clear recommendation of CABG as the gold standard in the treatment of significant ULMCA disease [7], [8], daily clinical practice demonstrates patients’ choice of PCI rather than CABG as the preferred strategy in the treatment of their significant ULMCA disease [9], [12]–[19]. Of importance is that not only are the outcomes of these randomized or non-randomized clinical studies [9], [12]–[19] consistent, the safety, feasibility, and profits of the use of PCI in this clinical setting are also notable. In fact, myriad reasons for patients with significant ULMCA disease to choose other management options other than CABG in the real word clinical practice have been reported [11], [16], [24], [25].

One important finding in the present study is that more than 300 patients who did not receive CABG due to various reasons (please see the inclusion and exclusion enrollment criteria) were willing to receive PCI for the treatment of their significant ULMCA disease within a period of less than 3 years. This implies that PCI may be an alternative treatment strategy in the treatment of patients with significant ULMCA obstruction in our daily clinical service.

Another significant finding is that the outcomes of angiography were excellent (100% procedural success) in various clinical settings in the current study. Our findings, therefore, highlight the reliability of PCI as a safe and feasible therapeutic option for patients with significant ULMCA disease unsuitable for CABG.

### Stenting to ULMCA Disease-Procedural and Clinical Success Rate in Low-Risk and High-Risk Patients

It is noteworthy that in the relatively low-risk subgroups (i.e., SAP and UAP), not only was the procedural success rate (100%) excellent, but the clinical success rate is also very promising (i.e., <2.8% of 30-day death). Previous clinical trials have shown that PCI treatment for significant ULMCA disease was associated with a very high successful rate with a very low incidence of 30-day major adverse cardiac event [12]–[19], [24], [25]. In this way, our findings are consistent with those of previous studies [12]–[19], [24], [25].

The most important finding in the present study is that the use of prophylactic IABP support (i.e., as a bridge to stenting) for patients with significant ULMCA disease and poor heart function undergoing stenting provided excellent angiographic results (i.e., 100% procedural success) and good 30-day clinical success (i.e., 0% mortality). Our findings provide important clinical information and encourage the use of this approach in the setting of significant ULMCA disease in our real word clinical practice.

In the current study, the 30-day mortality rate in the non-STEMI (i.e., high-risk) subgroup was around 8% after ULMCA stenting. Interestingly, one previous study has revealed that the 30-day mortality rate was 12% in patients with non-STEMI and significant ULMCA disease after receiving CABG [26]. Additionally, our previous study has shown that the 30-day mortality was notably high (15% mortality) in the high-risk subgroup of STEMI even after primary PCI [27]. Taken together, our results were not inferior to those of previous studies [26]. Furthermore, another previous study has shown that the in-hospital mortality was found to be remarkably high (i.e., >19.0%) in the high-risk subgroups of acute coronary syndrome (ACS) undergoing emergency CABG [28]. In this way our result was superior to that of the previous study [28].

### Stenting to ULMCA Disease-Procedural and Clinical Success Rate in Very High-Risk Patients

Of particular importance is that mechanical (i.e., IABP and/or ECMO)-assisted PCI for the high-risk patients showed 100% procedural success rate and acceptable 30-day clinical outcome [30-day mortality: 17.8% (8/45)]. Further analysis revealed that the 30-day cardiac-related death in this very high-risk patient subgroup was 11.1% (5/45). Therefore, the results of the present study were comparable to those of previous studies [27], [28].

### Stenting to ULMCA Disease-The 30-Day Cardiac-Associated Death and the Independent Predictors of 30-Day Mortality

The overall 30-day cardiac-related death was 2.9% (9/309) (Table 3-A). The acceptable lower 30-day mortality was in a similar clinical setting of ULMAC disease reported by the previously PCI or CABG clinical trials [6], [12]–[18].

In concert with the results of the previous studies that identified impaired renal function and acute renal failure as the strong predictors of short-term and long-term clinical outcome in ACS patients undergoing PCI [29]–[32], multivariate analysis identified acute kidney injury as the most powerful independent predictor of 30-day mortality in the current study. Additionally, WBC count, an index of inflammation, and increased BMI rather than the angiographic findings were another two in independently predictive of 30-day mortality. These findings suggest that successful PCI to ULMCA disease is no more the critical role for the poor prognostic outcome.

### Study Limitations

This study has limitations. First, the retrospective nature of this study cannot completely exclude the possibility of the presence of bias. Second, additional information on risk and prognostic stratification of ULMCA disease using PCI as a therapeutic option was not available without routine calculation of the SYNTAX score prior to or after PCI in our daily clinical practice. However, this study did not attempt to exclude any patient in our real world practice and in fact that our results were promising.

### Conclusion

The study presents high rates of procedural success and excellent 30-day clinical outcomes, without making assertions about safety.
